# Vitamin D suppresses bleomycin-induced pulmonary fibrosis by targeting the local renin–angiotensin system in the lung

**DOI:** 10.1038/s41598-021-96152-7

**Published:** 2021-08-16

**Authors:** Jianjun Chang, Hongguang Nie, Xin Ge, Jie Du, Weicheng Liu, Xue Li, Yue Sun, Xinzhi Wei, Zhe Xun, Yan Chun Li

**Affiliations:** 1 Institute of Health Sciences, China Medical University, Shenyang, Liaoning China; 2 Department of Physiology, China Medical University, Shenyang, Liaoning China; 3 Department of Stem Cells and Regenerative Medicine, College of Basic Medical Science, China Medical University, Shenyang, Liaoning China; 4 Department of Medicine, Division of Biological Sciences, The University of Chicago, Chicago, IL 60637 USA

**Keywords:** Diseases, Endocrinology, Pathogenesis

## Abstract

Idiopathic pulmonary fibrosis (IPF) is a severe disorder leading to progressive and irreversible loss of pulmonary function. In this study we investigated the anti-fibrotic effect of vitamin D using a mouse model of IPF. Lung fibrosis was induced with bleomycin in vitamin D-sufficient and vitamin D-deficient C57BL/6 mice. We found that treatment with active vitamin D analog paricalcitol prevented mouse body weight loss and alleviated lung fibrosis, whereas vitamin D deficiency severely aggravated lung injury. At the molecular level, paricalcitol treatment suppressed the induction of fibrotic inducer TGF-β and extracellular matrix proteins α-SMA, collagen type I and fibronectin in the lung, whereas vitamin D deficiency exacerbated the induction of these proteins. Interestingly, bleomycin treatment activated the local renin–angiotensin system (RAS) in the lung, manifested by the induction of renin, angiotensinogen, angiotensin II and angiotensin receptor type 1 (AT1R). Paricalcitol treatment suppressed the induction of these RAS components, whereas vitamin D deficiency enhanced the activation of the lung RAS. We also showed that treatment of bleomycin-induced vitamin D-deficient mice with AT1R antagonist losartan relieved weight loss, substantially ameliorated lung fibrosis and markedly blocked TGF-β induction in the lung. Moreover, we demonstrated that in lung fibroblast cultures, TGF-β and angiotensin II synergistically induced TGF-β, AT1R, α-SMA, collagen type I and fibronectin, whereas 1,25-dihydroxyvitamin D markedly suppressed the induction of these fibrotic markers. Collectively, these observations strongly suggest that vitamin D mitigates lung fibrosis by blocking the activation of the lung RAS in this mouse model of IPF.

## Introduction

Idiopathic pulmonary fibrosis (IPF) is a chronic and progressive interstitial lung disease^1,2^ that is characterized by proliferation of fibroblasts and reconstruction of collagen (Col)-based extracellular matrix (ECM)^3,4^. Repetitive local injuries to an ageing alveolar epithelium is thought to play a key role in the process of IPF, which result in premature and persistent senescence of epithelial cells, excessive generation of pro-fibrotic mediators and sustained activation of mesenchymal cells, leading to the development of IPF^1^. The increases in the rates of hospital admissions and deaths resulting from IPF indicate an increasing burden of this devastating disease^5,6^. Bleomycin (BLM)-induced lung fibrosis in mice is a well-accepted experimental model of IPF for IPF research^7^. Despite great advances in this research area, the molecular basis of IPF remains incompletely understood.

The vitamin D endocrine system plays pleiotropic roles in the physiology and pathophysiology of humans and animals^8^. The vitamin D hormone, 1,25-dihydroxyvitamin D (1,25(OH)_2_D_3_), interacts with the vitamin D receptor (VDR) to exert biological activities. The classic function of vitamin D is to regulate calcium homeostasis and skeletal health, but numerous non-classic functions of vitamin D have been reported in recent years including regulation of cell proliferation and anti-oxidative and anti-inflammatory effects^9–12^. One important finding is that the vitamin D hormone is a negative endocrine regulator of the renin–angiotensin system (RAS)^13^. The RAS plays key roles in the regulation of blood pressure and salt and fluid balances, but the local RAS within different tissues in the body have various biological activities^14^. Renin is the rate-limiting enzyme that cleaves angiotensinogen (AGT) to angiotensin (Ang) I, which is further processed to Ang II by angiotensin-converting enzyme (ACE). Ang II, the central effector of the RAS, is well known to have potent pro-inflammatory and pro-fibrotic activities, which are mediated by angiotensin receptor type 1 (AT1R)^15,16^. High ACE levels were reported in the broncho-alveolar fluid in fibrotic lung diseases^17^, and AGT is one of the most overexpressed genes in pulmonary fibrosis patients^18^. In fact, our previous studies demonstrated that activation of the RAS dramatically promotes lung fibrosis in mice^19^.

The VDR is highly expressed in the lung, suggesting that the lung is a non-classic target organ of vitamin D actions in the body^20^. Epidemiological data have suggested an association between vitamin D deficiency (VDD) and increased risks of infections in lungs^21,22^. VDD is common in patients with acute respiratory distress syndrome^23^. Low level of 25(OH)D is associated with pulmonary exacerbations in patients with cystic fibrosis^24^. VDD is also positively associated with the mortality of IPF patients^25^. In mice chronic VDD leads to lung fibrosis^26^, and our previous studies demonstrated that VDR-deficiency promotes acute lung injury due to the activation of the local RAS and Ang-2-Tie-2 pathways in the lung^27^. It was also reported that vitamin D attenuates BLM-induced IPF by inhibiting myofibroblast proliferation^28^. Based on these observations, we hypothesized that the local RAS in the lung is a key target of vitamin D in vitamin D prevention of BLM-induced lung injury. In this report we presented evidence from vitamin D analog therapy and a VDD model to support this hypothesis.

## Results

### Paricalcitol attenuates BLM-induced lung fibrosis in mice

**Figure 1:**
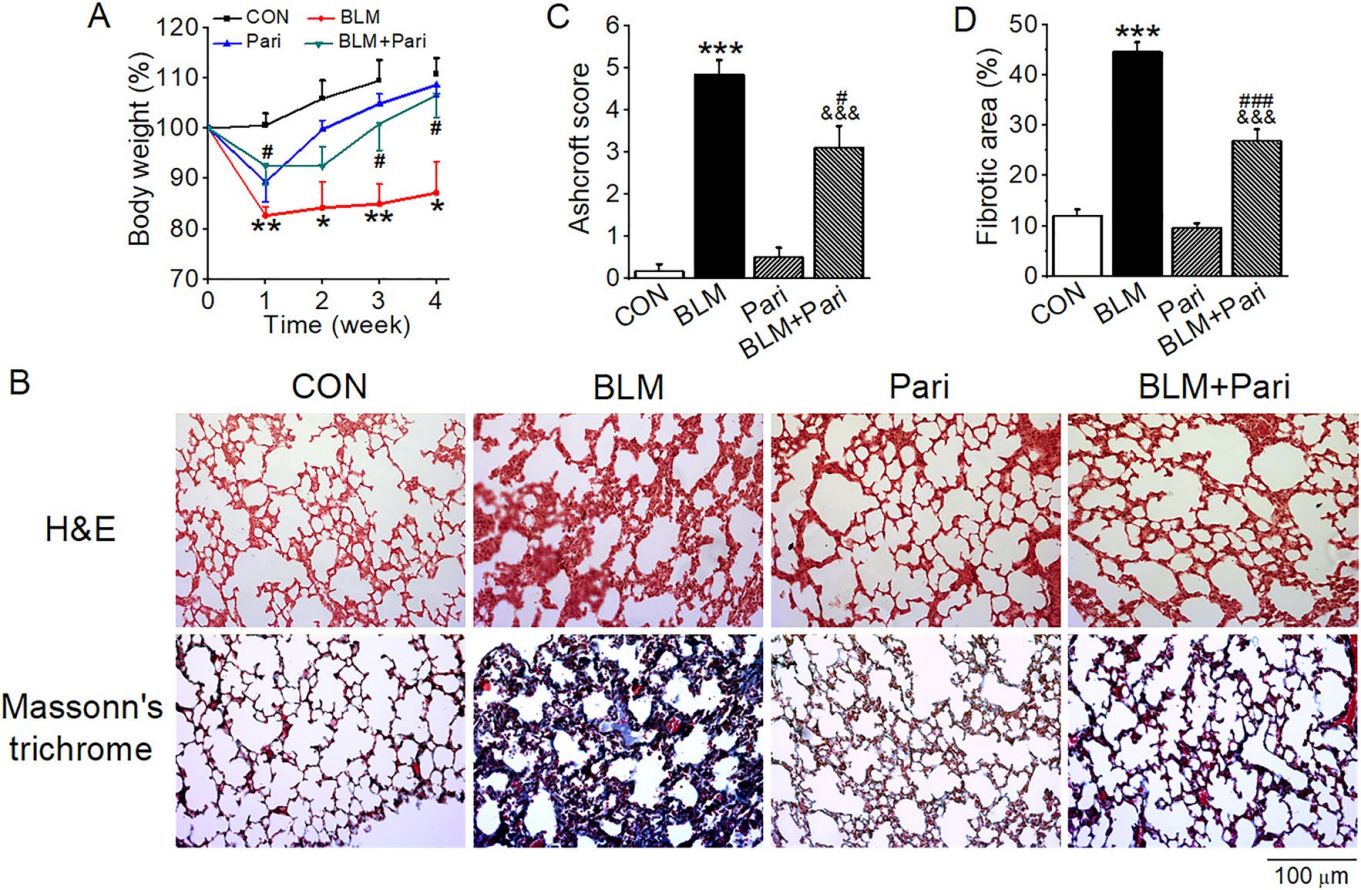
Paricalcitol attenuates BLM-induced structural damage and lung fibrosis in mice. (**A**) Mouse body weight changes in the course of BLM induction. *P < 0.05, **P < 0.01, versus CON group; ^#^*P* < 0.05, versus BLM group; by two-way repeated measures ANOVA. *n* = 4–5. (**B**) H&E and Masson’s trichrome staining of lung sections. Original magnification: 200 × . (**C**) Microscopic scores of the lung sections according to Ashcroft’s method. ***P < 0.001, versus CON group; ^&&&^*P* < 0.001, versus Pari group; ^#^*P* < 0.05, versus BLM group, by two-way ANOVA. *n* = 6–8. (**D**) Quantification of collagen-positive areas based on Masson’s trichrome staining. ***P < 0.001, versus CON group; ^&&&^*P* < 0.001, versus Pari group; ^###^*P* < 0.001, versus BLM group, by two-way ANOVA. *n* = 6–8 each group. CON, control; BLM, bleomycin; Pari, paricalcitol.

To explore the therapeutic effect of vitamin D on BLM-induced lung injury, we treated BLM-induced IPF model with paricalcitol, a low-calcemic VDR agonist. As shown in Fig. 1, intratracheal instillation of BLM resulted in marked body weight loss that was not recovered in the following 4 weeks, but paricalcitol was able to prevent the weight loss and actually promoted weight gain in BLM-induced mice (Fig. 1A). Mice receiving paricalcitol treatment had some weight loss initially, probably due to hypercalcemic effects. Histological examination revealed destructive alveolar structure and thickened alveolar septum (H&E staining) and severe interstitial ECM deposition (Masson trichrome staining) in the lung of BLM-induced mice, and paricalcitol treatment markedly mitigated these events (Fig. 1B). Consistently, semi-quantitative analyses showed BLM dramatically increased the Ashcroft score, which measures lung fibrosis, and the fibrotic areas in the lung, whereas paricalcitol significantly attenuated the increase of these parameters (Fig. 1C,D).

**Figure 2:**
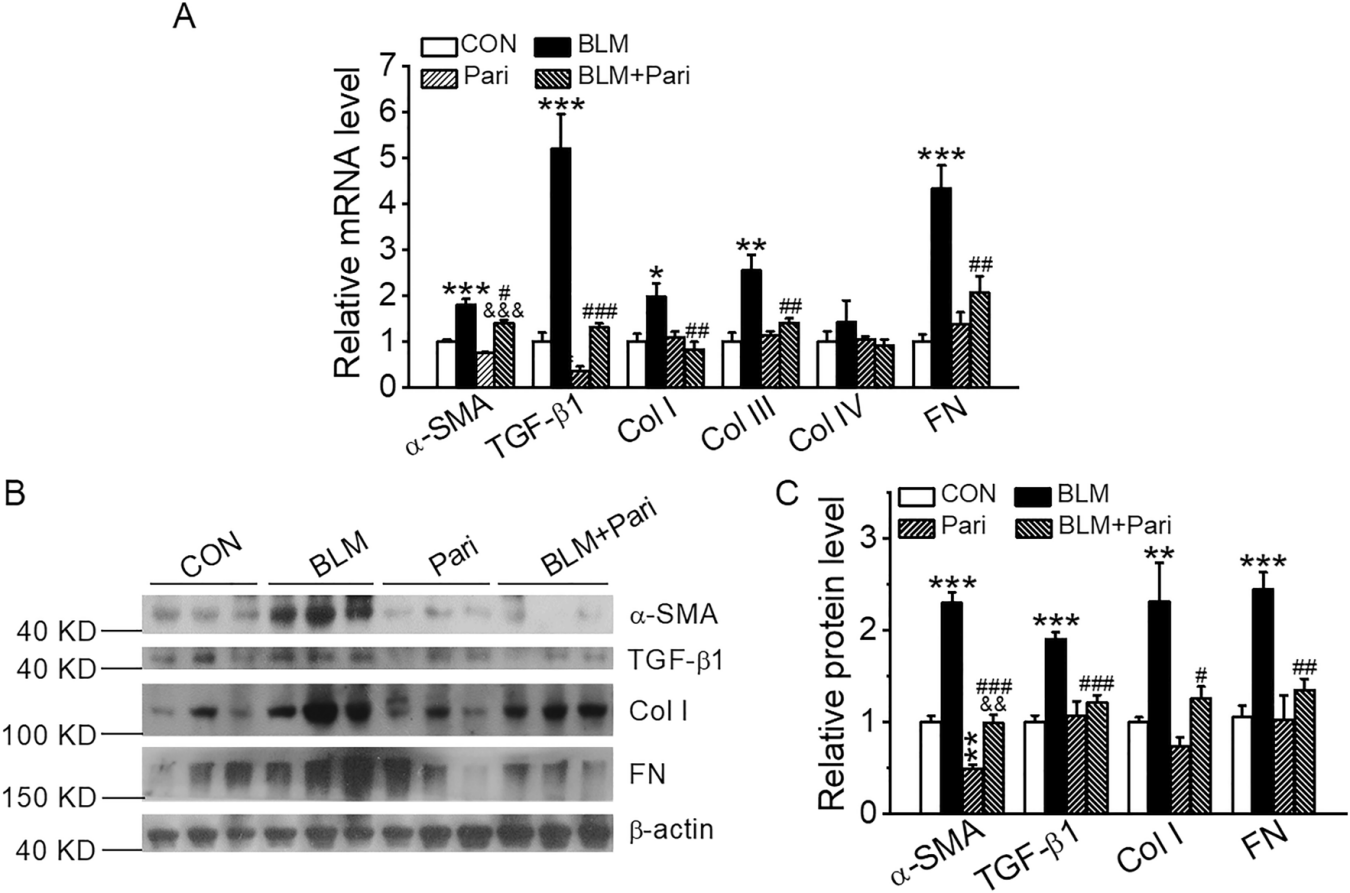
Paricalcitol blocks BLM induction of pro-fibrotic proteins in the lung. (**A**) Real-time RT-PCR quantitation of mRNA expressions of pro-fibrotic genes. *P < 0.05, **P < 0.01, ***P < 0.001, versus CON group; ^&&&^*P* < 0.001, versus Pari group; ^#^*P* < 0.05, ^##^*P* < 0.01, ^###^*P* < 0.001, versus BLM group; by two-way ANOVA and Kruskal–Wallis one-way ANOVA (for Col I levels). *n* = 3–4. (**B**,**C**) Western blot analysis (**B**) and densitometric quantitation (**C**) of α-SMA, TGF-β1, Col I and FN proteins in the lung lysates. **P < 0.01, ***P < 0.001, versus CON group; ^&&^*P* < 0.01, versus Pari group; ^#^*P* < 0.05, ^##^*P* < 0.01, ^###^*P* < 0.001, versus BLM group; by two-way ANOVA. *n* = 5–7 each group.

We next investigated the expression of fibrosis-related genes by quantitative RT-PCR and Western blotting. As expected, fibrogenic factor TGF-β1 and ECM proteins, including α-smooth muscle actin (SMA), Col I, Col III, Col IV and fibronectin (FN), were markedly induced in BLM-induced lungs at both mRNA (Fig. 2A) and protein (Fig. 2B,C) levels, and paricalcitol treatment substantially blocked the induction of these proteins at both mRNA and protein levels (Fig. 2A–C).

### Vitamin D deficiency aggravates BLM-induced lung injury

**Figure 3:**
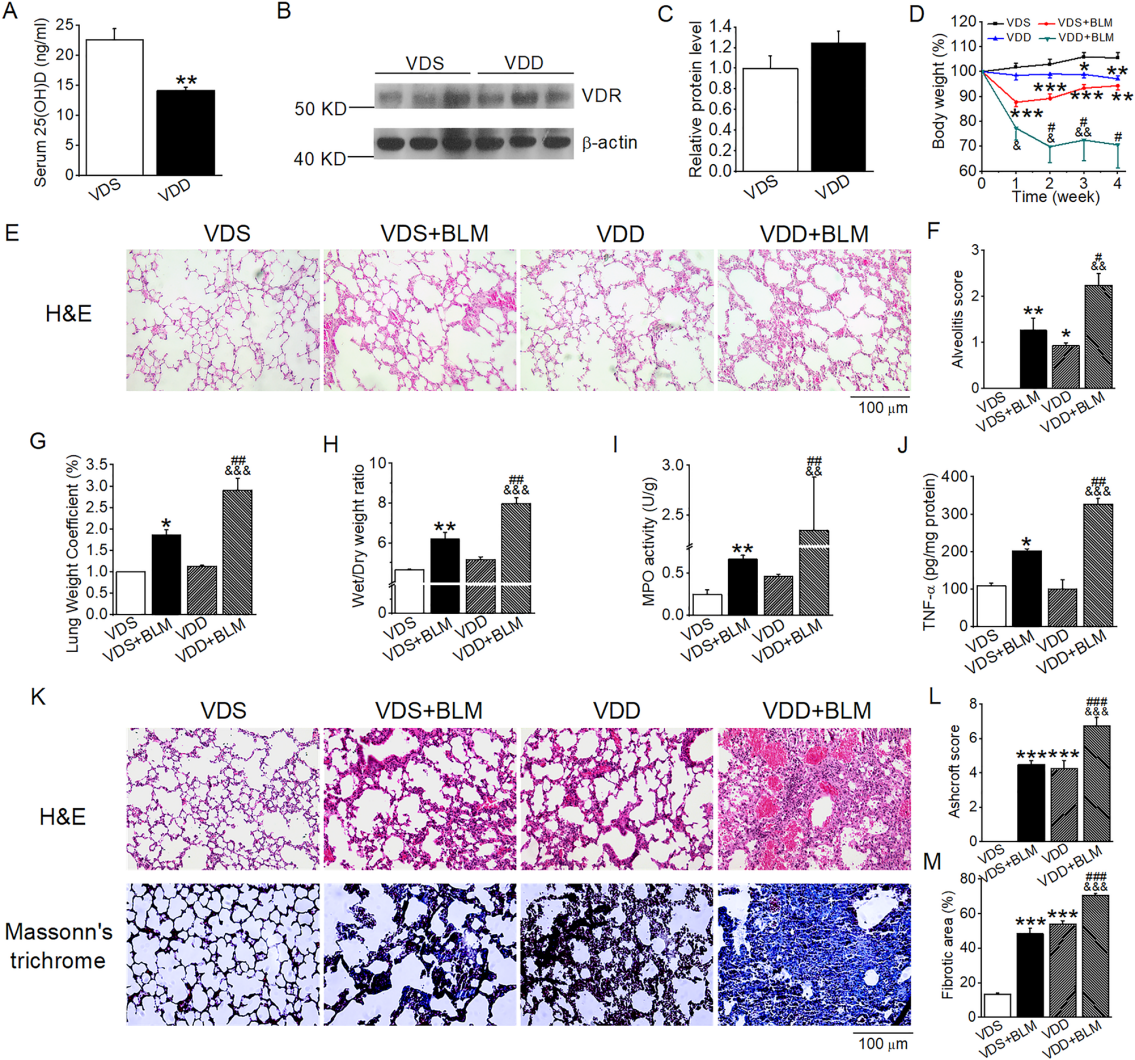
Vitamin D deficiency exacerbates BLM-induced structural damage and lung fibrosis in mice. (**A**) Serum 25(OH)D levels in mice fed VDS or VDD diets for 9 weeks. **P < 0.01, versus VDS group. *n* = 5 each group. (**B**,**C**) Western blot analysis (**B**) and densitometric quantitation (**C**) of pulmonary vitamin receptor (VDR) in VDS and VDD mice. *n* = 6–7 each group. (**D**) Mouse body weight changes in the course of 4 weeks after BLM induction. *P < 0.05, **P < 0.01, ***P < 0.001, versus VDS group; ^&^*P* < 0.05, ^&&^*P* < 0.01 versus VDD group; ^#^*P* < 0.05, versus VDS + BLM group; by two-way repeated measures ANOVA. *n* = 5–6 each group. (**E**) H&E staining of the lung sections 2 weeks after BLM induction. Original magnification: 200×. (**F**) Alveolitis scores of the lung sections. (**G**) Lung weight coefficient. (**H**) Wet/dry weight ratio. (**I**) TNF-α levels in lung lysates. (**J**) Myeloperoxidase (MPO) levels in lung lysates. *P < 0.05, **P < 0.01, versus VDS group; ^&&^*P* < 0.01, ^&&&^*P* < 0.001 versus VDD group; ^#^*P* < 0.05, ^##^*P* < 0.1 versus VDS + BLM group; by two-way ANOVA. *n* = 3–4 each group. (**K**) H&E and Masson’s trichrome staining of lung Sects. 4 weeks after BLM induction. Original magnification: 200×. (**L**) Microscopic scores of lung sections according to Ashcroft’s method. (**M**) Quantification of Col-positive areas based on Masson’s trichrome staining. ***P < 0.001, versus VDS group; ^&&&^*P* < 0.001, versus VDD group, ^###^*P* < 0.001, versus VDS + BLM group; by Kruskal–Wallis one-way ANOVA. *n* = 4–6 each group.

**Figure 4:**
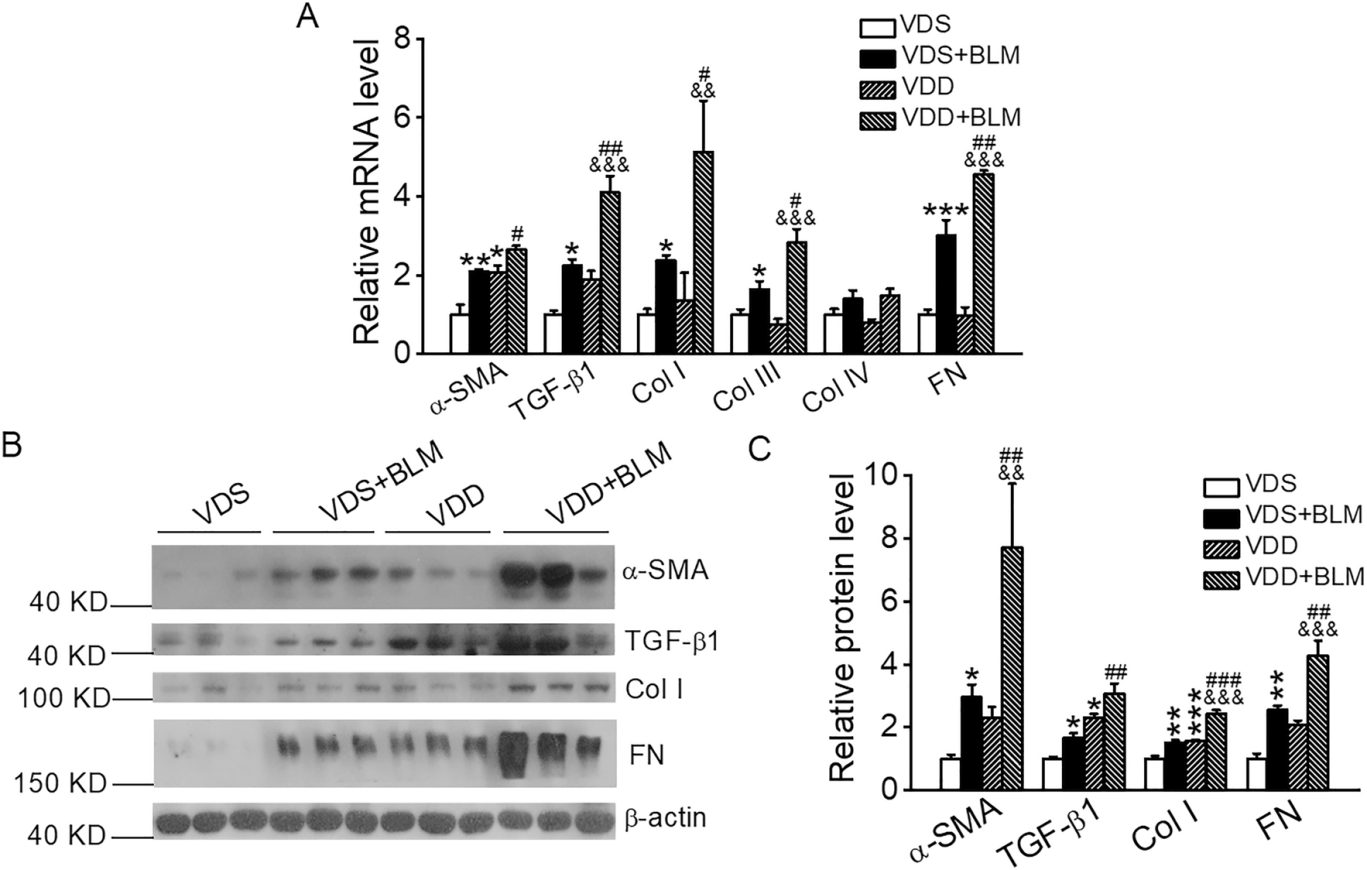
Vitamin D deficiency aggravates the induction of pro-fibrotic factors by BLM. (**A**) Real-time RT-PCR quantitation of mRNA expressions of pro-fibrotic genes. *P < 0.05, **P < 0.01, ***P < 0.001, versus VDS group; ^&&^*P* < 0.05, ^&&&^*P* < 0.001, versus VDD group, ^#^*P* < 0.05, ^##^*P* < 0.01, versus VDS + BLM group; by two-way ANOVA. *n* = 3–4 each group. (**B**,**C**) Western blot analysis (**B**) and densitometric quantitation (**C**) of α-SMA, TGF-β1, Col I and FN in the lung lysates. *P < 0.05, **P < 0.01, ***P < 0.001, versus VDS group; ^&&^*P* < 0.05, ^&&&^*P* < 0.001, versus VDD group; ^##^*P* < 0.01, ^###^*P* < 0.001, versus VDS + BLM group; by two-way ANOVA and Kruskal–Wallis one-way ANOVA (for α-SMA levels). *n* = 5–6 each group.

To further investigate the role of vitamin D in the development of IPF, we next explored the effect of VDD on BLM-induced lung injury. VDD model was established by placing the mice on a VDD diet for 9 weeks, in which the serum 25(OH)D level was significantly decreased to less than 15 ng/ml in these mice (Fig. 3A). Western blot assay revealed that lung VDR expression was not significantly changed in VDD mice compared with vitamin D-sufficient (VDS) mice (Fig. 3B,C). Following BLM insult, VDD mice exhibited much more severe weight loss in the next 4 weeks compared with VDS mice (Fig. 3D). Histological examination revealed that the VDD mice already developed marked lung injury 2 weeks after BLM induction, manifested by increased thickening of the alveolar wall and marked increases in the alveolar interstitial space (Fig. 3E), suggesting a development of pulmonary edema. Consistently, at this time the VDD mice had much higher Alveolitis score (Fig. 3F), lung weight coefficient (Fig. 3G) and wet/dry weight ratio (Fig. 3H) compared with the VDS mice. Furthermore, the levels of MPO and TNF-α in the lung lysates were also higher in the VDD mice than in the VDS mice (Fig. 3I,J). As expected, at 4 weeks following BLM instillation, lung injury became even more severe in the VDD mice compared with the VDS counterparts, manifested by disarrangement of alveolar architecture, very severe interstitial fibrosis, intra-alveolar hemorrhage and massive inflammatory cell infiltration (Fig. 3K). Quantitative data for the Ashcroft score (Fig. 3L) and fibrotic areas (Fig. 3M) in the lung were consistent with the histological observations. Interestingly, the VDD mice without BLM induction also developed lung injury comparable to the VDS + BLM mice (Fig. 3K–M), which is consistent with a previous report showing VDD induced lung fibrosis^26^. This observation underlines the important protective role of vitamin D in the lung. Further analyses of the molecular biomarkers confirmed that VDD exacerbated BLM induction of TGF-β1, α-SMA, Col I, Col III, Col IV and FN in the lung at both the mRNA and protein levels, compared with the VDS counterparts (Fig. 4A–C).

### Vitamin D modulates the local RAS to suppress BLM-induced lung fibrosis

**Figure 5:**
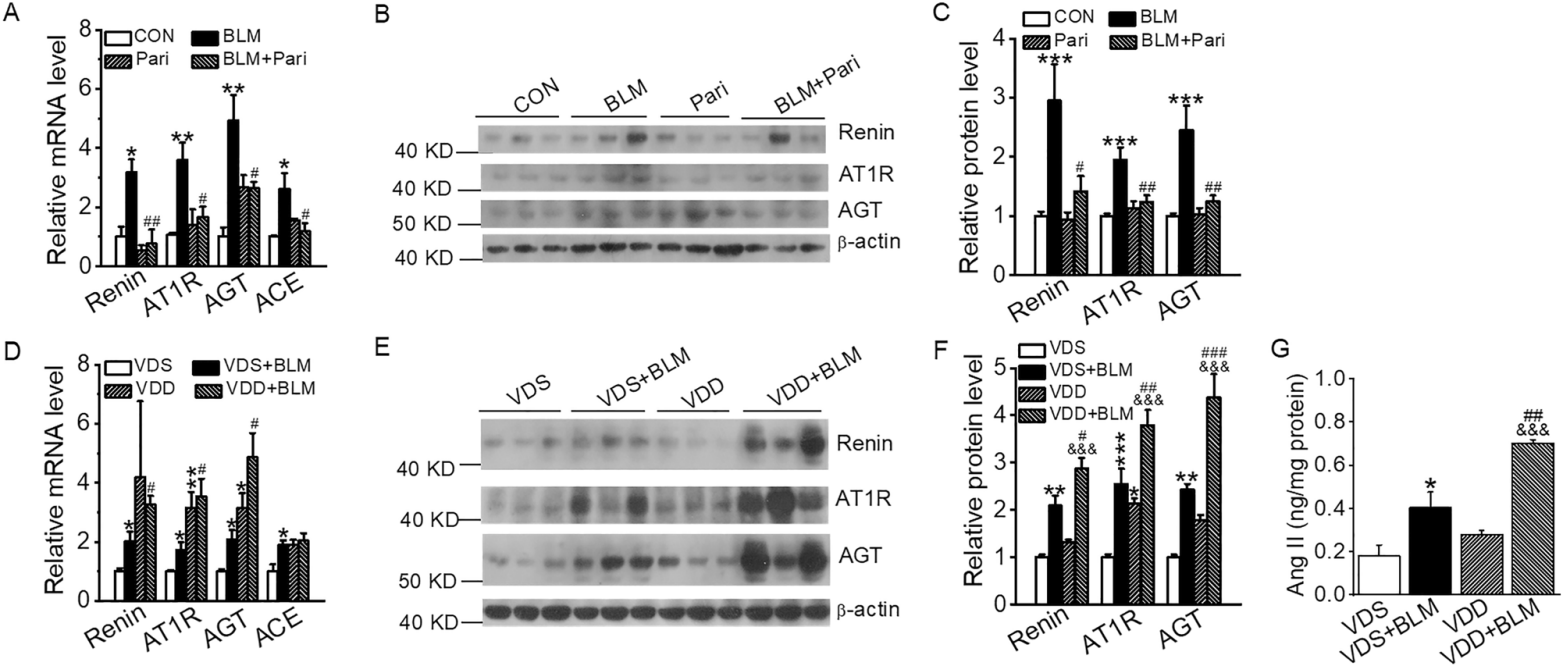
Vitamin D targets the pulmonary renin–angiotensin system to suppress BLM-induced lung fibrosis. (**A**) Relative mRNA levels of RAS components in paricalcitol-treated BLM-induced mice. *P < 0.05, **P < 0.01, versus CON group; ^#^*P* < 0.05, ^##^*P* < 0.01, versus BLM group; by two-way ANOVA. *n* = 3–4 each group. (**B**,**C**) Western blot (**B**) and densitometric quantitation (**C**) of renin, angiotensin type 1 receptor (AT1R), and angiotensinogen (AGT) in paricalcitol-treated BLM-induced mice. ***P < 0.001, versus CON group; ^#^*P* < 0.05, ^##^*P* < 0.01, versus BLM group; by two-way ANOVA and Kruskal–Wallis one-way ANOVA (for AT1R levels). *n* = 5–7. (**D**) Relative mRNA expressions of RAS components in BLM-induced VDS and VDD mice. *P < 0.05, **P < 0.01, versus VDS group; ^#^*P* < 0.05, versus VDS + BLM group; by two-way ANOVA. *n* = 3–4 each group. (**E**,**F**) Western blot (**E**) and densitometric quantitation (**F**) of renin, AT1R and AGT in BLM-treated VDS and VDD mice. *P < 0.05, **P < 0.01, ***P < 0.001, versus VDS group; ^&&&^*P* < 0.001, versus VDD group; ^#^*P* < 0.05, ^##^*P* < 0.01, ^###^*P* < 0.001, versus VDS + BLM group; by two-way ANOVA. *n* = 6–8 each group. (**G**) Quantitation of Ang II levels in lung lysates. *P < 0.05, versus VDS group; ^&&&^*P* < 0.001, versus VDD group; ^##^*P* < 0.01, versus VDS + BLM group; by two-way ANOVA. *n* = 5–7 each group.

Given the role of the RAS in lung injury revealed by our previous studies^19,27^, we examined the status of the local RAS in the lung from the BLM-induced mice. Quantitative analyses using real time RT-PCR and Western blot assays showed that the components of the RAS cascade, including renin, ACE, AGT and AT1R, were induced in the lung of BLM-treated mice at both the mRNA (Fig. 5A,D) and protein levels (Fig. 5B,C,E,F). Paricalcitol treatment significantly blocked these inductions (Fig. 5A–C), whereas VDD further enhanced these BLM-induced up-regulations (Fig. 5D–F). Importantly, lung Ang II contents in BLM-induced mice were significantly increased (Fig. 5G), a crucial indicator of RAS activation. These observations strongly suggest that the local RAS in the lung is activated by BLM, and the vitamin D signaling modulates the lung RAS to ameliorate BLM-induced lung injury in these mice.

### Blockade of RAS activation alleviates BLM-induced lung fibrosis in VDD mice

**Figure 6:**
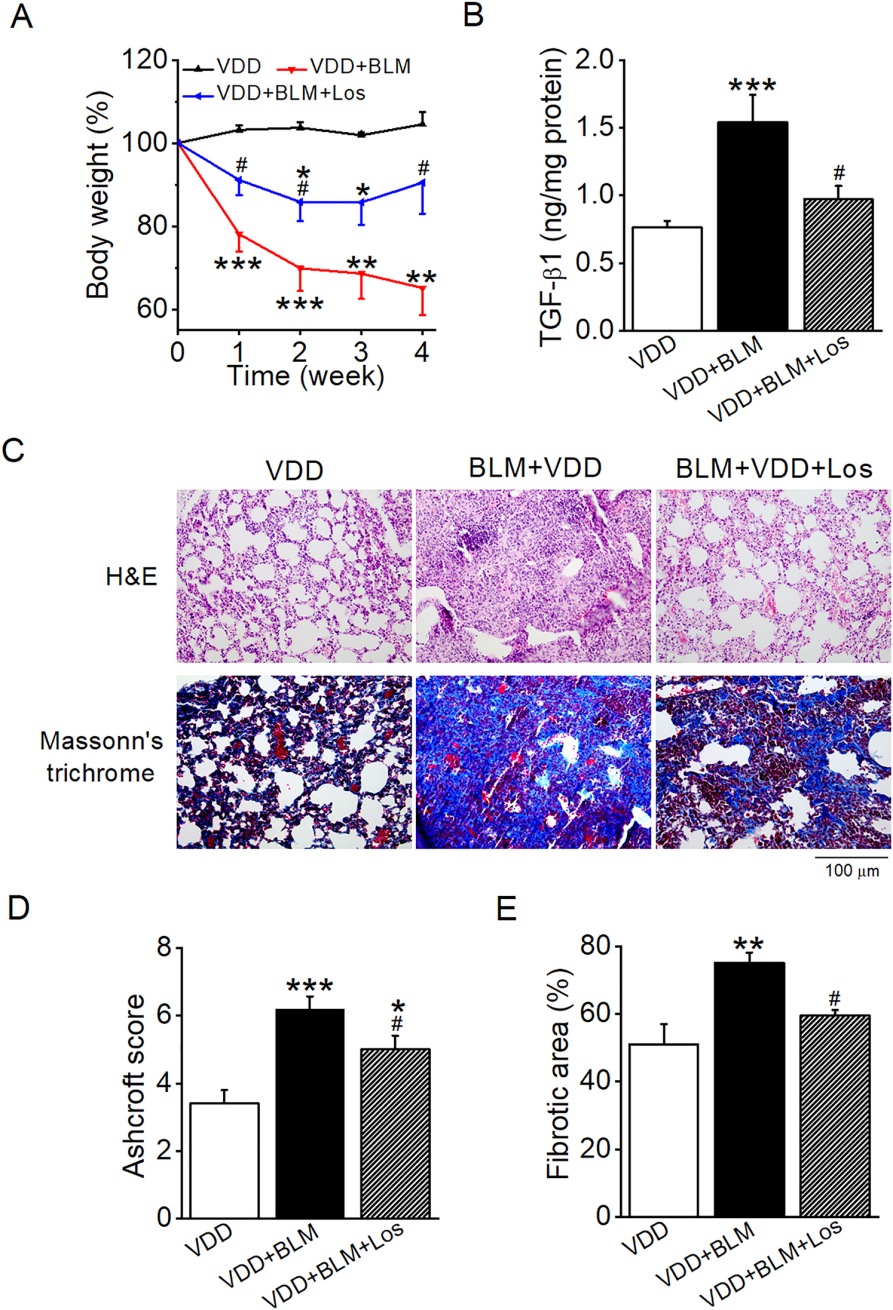
Blockade of RAS activation alleviates BLM-induced lung fibrosis in VDD mice. (**A**) Body weight changes in the course of 4 weeks of BLM induction with or without losartan treatment. *P < 0.05, **P < 0.01, ***P < 0.001 versus VDD group; ^#^*P* < 0.05, versus VDD + BLM group; by two-way repeated measures ANOVA. *n* = 5–7 each group. (**B**) TGF-β1 levels in lung lysates. ***P < 0.001, versus VDD group; ^#^*P* < 0.05, versus VDD + BLM group; by one-way ANOVA. *n* = 4–7 each group. (**C**) H&E and Masson’s trichrome staining of lung sections. (**D**) Microscopic scores of lung sections according to Ashcroft’s method. (**E**) Quantification of collagen-positive areas based on Masson’s trichrome staining. *P < 0.05, **P < 0.01, ***P < 0.001, versus VDD group; ^#^*P* < 0.05, versus VDD + BLM group; by one-way ANOVA. *n* = 4–6 each group. Los, losartan.

To confirm the role of RAS in the VDD-aggravated lung injury, we treated BLM-induced VDD mice with an AT1R blocker, losartan. We focused on VDD mice for this experiment because these mice showed much robust induction of the lung RAS, suggesting that the local RAS contributes more to lung injury in the VDD mice compared with the VDS mice. Therefore, the VDD mice are more relevant to test the effect of RAS inhibitors. As shown in Fig. 6, losartan treatment significantly attenuated body weight loss of BLM-induced VDD mice (Fig. 6A). Losartan also significantly blocked TGF-β1 induction in the lung of BLM-induced VDD mice (Fig. 6B). At the histological level, losartan partially restored the collapsed alveoli and dramatically decreased the interstitial ECM deposition in the lung seen in BLM-induced VDD mice (Fig. 6C). The Ashcroft score and lung fibrotic area were also significantly improved in losartan-treated mice (Fig. 6D,E). These observations suggest that VDD aggravates BLM-induced lung fibrosis due to overactivation of the local RAS.

### Vitamin D suppresses Ang II-induced pro-fibrotic biomarkers in mouse lung fibroblasts

**Figure 7:**
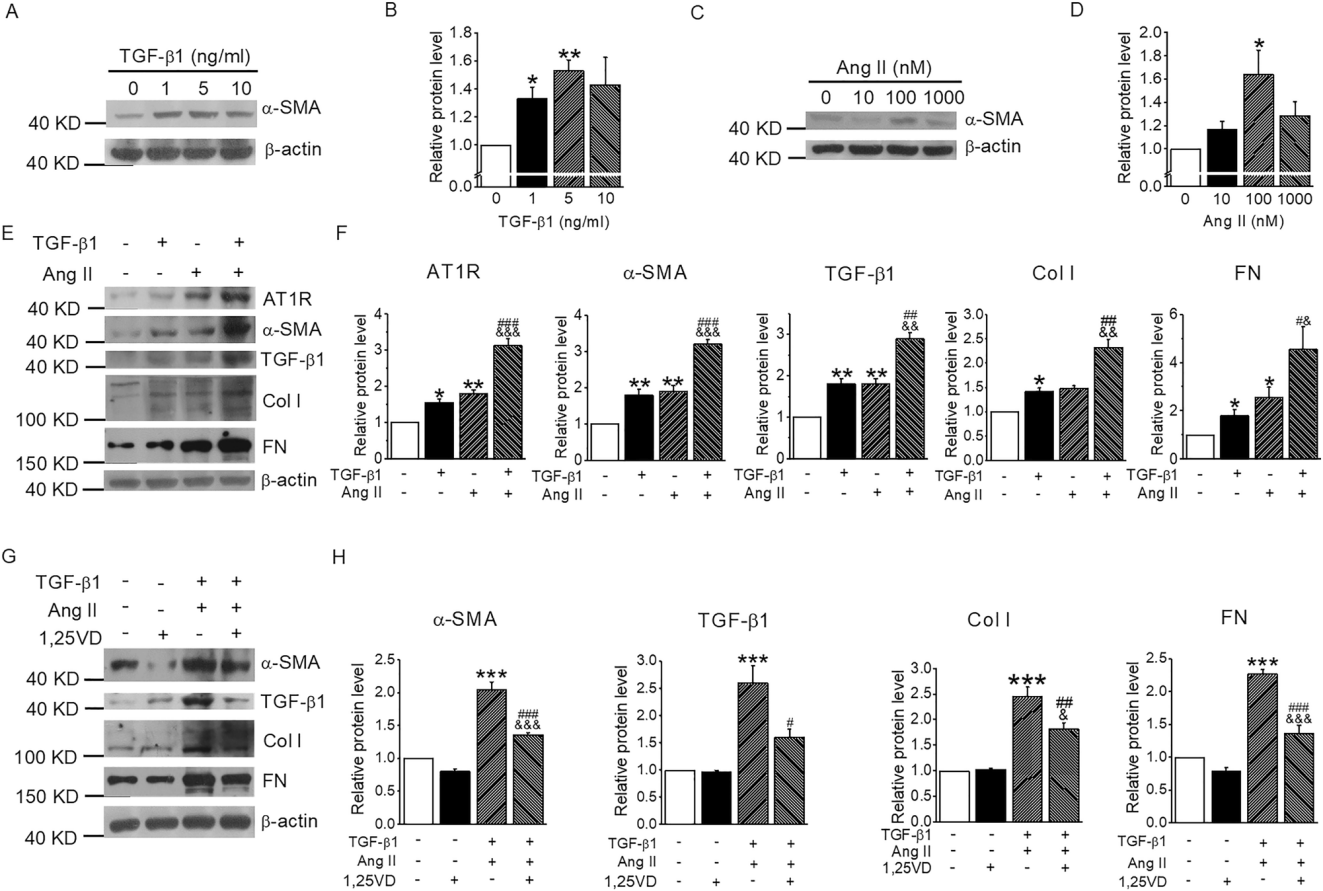
1,25(OH)_2_D_3_ Vitamin D suppresses Ang II-induced pro-fibrotic biomarkers in mouse lung fibroblasts. (**A**,**B**) Western blot (**A**) and densitometric quantitation (**B**) of α-SMA in MLg2908 cells treated by TGF-β1 (0, 1, 5, 10 ng/ml) for 24 h. *P < 0.05, **P < 0.01, versus 0 group; by one-way ANOVA. *n* = 3. (**C**,**D**) Western blot (**C**) and densitometric quantitation (**D**) of α-SMA in MLg2908 cells treated by Ang II (0, 10, 100, 1000 nM) for 24 h. *P < 0.05, versus 0 group; by one-way ANOVA. *n* = 3. (**E**,**F**) Western blot (**E**) and densitometric quantitation (**F**) of pro-fibrotic proteins in MLg2908 cells treated by 5 ng/ml TGF-β1, 100 nM Ang II or both for 24 h. *P < 0.05, **P < 0.01, versus control group; ^&^*P* < 0.05, ^&&^*P* < 0.01, ^&&&^*P* < 0.001, versus Ang II group; ^#^*P* < 0.05, ^##^*P* < 0.01, ^###^*P* < 0.001, versus TGF-β1 group; by two-way ANOVA. *n* = 3–4. (**G**,**H**) Western blot (**G**) and densitometric quantitation (**H**) of pro-fibrotic proteins in MLg2908 cells treated by both TGF-β1 and Ang II in the presence or absence of 1,25(OH)2D3. ***P < 0.001, versus control group; ^&^*P* < 0.05, ^&&&^*P* < 0.001, versus 1,25VD group; ^#^*P* < 0.05, ^##^*P* < 0.01, ^###^*P* < 0.001, versus TGF-β1 + Ang II group; by two-way ANOVA. *n* = 5–7.

Ang II and TGF-β1 are potent fibrogenic factors and both exist in the lung of BLM-induced mice (Figs. 2, 4, 5). We confirmed that both TGF-β1 and Ang II were able to induce fibrotic marker α-SMA in lung MLg2908 fibroblast cells in a dose-dependent manner (Fig. 7A–D). Importantly, combining TGF-β1 and Ang II together at the optimal doses (5 ng/ml and 100 nM, respectively) maximized the induction of AT1R, α-SMA, TGF-β1, Col I and FN in these cells, likely in a synergistic manner (Fig. 7E,F), confirming their powerful pro-fibrotic activities when these two factors are present together in the lung. Interestingly, when the cells were pre-treated with 1,25(OH)_2_D_3_ (20 nM), the active hormone of vitamin D, the pro-fibrotic activities of the TGF-β1 and Ang II combination were dramatically attenuated (Fig. 7G,H). Since RAS activation also induces TGF-β1, these data confirm that the 1,25(OH)_2_D_3_/VDR signaling in the lung protects against lung fibrosis induced by the activation of the RAS.

## Discussion

Pulmonary fibrosis represents an end stage of lung diseases and its development includes several distinct stages—clotting/coagulation, inflammation, fibroblast migration/proliferation/activation and tissue remodeling^29,30^. It is believed that at the inflammatory phase, impaired epithelial or endothelial cells produce inflammatory mediators that promote infiltration of inflammatory cells, which release inflammatory and pro-fibrotic factors that induce myofibroblast activation and interstitial ECM deposition^31–34^. The BLM-induced lung fibrosis model recapitulates many aspects of IPF, in which inflammatory infiltrates play critical roles in promoting ECM deposition and lung fibrosis^35–37^. In this study, we found that the local RAS cascade in the lung is another strong mediator of lung fibrosis in BLM-induced lung fibrosis model, especially under vitamin D deficiency. Renin, the rate-limiting enzyme of this cascade, is highly induced in the lung following BLM induction, but it is unclear whether renin is produced from the lung epithelial or endothelial cells or from inflammatory infiltrate cells, as mast cells and macrophages are rich sources of renin^38–40^. AGT, the substrate of renin, and ACE are both highly expressed in the lung^17,18^. All these provide a favorable environment for the lung to produce excess Ang II under inflammation and/or fibrosis. As a potent pro-fibrotic factor, Ang II acts on AT1R, which is also highly induced in the lung under fibrogenic conditions^41^, to promote TGF-β1 expression and lung fibrogenesis. TGF-β1 as a most powerful profibrogenic factor can induce fibroblast proliferation, transform fibroblasts into myofibroblasts and stimulate ECM synthesis^42^. Activated myofibroblasts deposit ECM, causing thickening of the alveolar walls^16^. We and others have reported that chronic activation of RAS promotes pulmonary fibrosis leading to lung dysfunction in mice^19,43^. In fact, our data presented in the current study confirmed this scenario of molecular events developed in the lung. We reported here that the RAS is highly activated in the BLM-induced lung fibrosis model, and blockade of the RAS with an AT1R antagonist losartan is able to ameliorate BLM-induced lung fibrosis.

Another important finding of this work is that vitamin D signaling protects against BLM-induced lung fibrosis via targeting the RAS. We reported that treatment with vitamin D analog paricalcitol attenuated BLM-induced lung fibrosis in a mouse IPF model, and the vitamin D hormone 1,25(OH)_2_D_3_ suppressed the expression of fibrogenic factors and ECM maker proteins in lung fibroblast cell cultures, whereas vitamin D deficiency further aggravated BLM-induced lung fibrosis in mice. All these observations are related to the RAS. This is not surprising, as a close relationship between vitamin D and RAS has been well established and the concept of vitamin D suppressing RAS activation has been well accepted^8,13,44,45^. Physiologically, the vitamin D hormone functions as a negative regulator of renin gene expression, and VDR deletion in mice led to hyperreninemia and hypertension^13^. Conversely, vitamin D or vitamin D analog compounds are able to directly suppress renin gene expression^46–48^.

A body of previous research has linked vitamin D to lung biology and pathophysiology and potent anti-fibrosis activity of vitamin D has been well documented. Vitamin D deficiency is closely associated with multiple lung diseases, including asthma, cystic fibrosis, interstitial lung diseases, chronic obstructive pulmonary disease and respiratory infections^49–51^. That vitamin D deficiency promotes tissue fibrosis has been reported in various organs, including the liver^52^, kidney^53^ and intestine^54^. In patients with cystic fibrosis, vitamin D deficiency was associated with increased pulmonary exacerbations and decreased lung function^24^. In a mouse model of acute lung injury, VDR deficiency exacerbated lung injury following lipopolysaccharide challenge, leading to higher mortality^27^. In a unilateral ureter obstruction model of kidney fibrosis, VDR deletion aggravated renal damage in the obstructed kidney and promoted interstitial fibrosis due to overactivation of the local RAS in the kidney^55^. Although a prior study reported that nutritional vitamin D supplementation was able to attenuate pulmonary fibrosis in the BLM-induced lung fibrosis model, the underlying molecular basis was not identified^28^. In the present study, we showed that key components of the RAS including renin, AGT and AT1R are highly induced under this condition, especially the production of Ang II in the lung is increased, suggesting that VDD-induced activation of the local RAS is at least attributed to the development of lung fibrosis under BLM induction. Indeed, the data from the losartan treatment experiments and in vitro data from MLg2908 cells lend strong support to this notion. Therefore, we conclude that vitamin D/VDR signaling suppresses BLM-induced lung fibrosis by inhibiting the activation of the local RAS in the lung. The therapeutic implication of this conclusion is that low calcemic vitamin D analogs and anti-RAS drugs may be useful for the management of human IPF.

## Materials and methods

### Animals

Three- and eight-week old male and female C57BL/6 mice were provided by the Laboratory Animal Center of China Medical University. The mice were housed in a pathogen-free facility, and maintained in a 12 h/12 h light/dark cycle at 25 °C temperature. Approximately equal numbers of male and female mice were used in each experiment. All experiments were performed according to the guidelines and regulations of the Animal Care and Use Ethics Committee of China Medical University, and all experimental protocols were approved by China Medical University. All animal studies were carried out in accordance with the ARRIVE guidelines.

### Paricalcitol treatment of BLM-induced IPF model

A mouse model of IPF was induced using BLM. Although a prior study has suggested that male sex contributes to the severity of BLM-induced pulmonary fibrosis in BLM model^56^, we did not observe clear differences in sex-related lung injury. Therefore, both male and female mice in approximately equal numbers were used in BLM treatment. Briefly, 8-week old mice were anesthetized and administered intratracheally with one dose of BLM (1.5 Units/kg, Sigma-Aldrich, St. Louis, MO) or vehicle on day 0. Vitamin D analog paricalcitol (150 ng/kg, dissolved in 70% propylene glycol) or vehicle was injected intraperitoneally on day 2, and paricalcitol treatment was continued every other day until the end of the experiment. Body weight was monitored daily. On day 28, the mice were sacrificed and lungs were harvested for analysis.

### BLM-induced IPF model in vitamin D-deficient mice and losartan treatment

To induce vitamin D deficiency, 3-week old mice were placed on a vitamin D deficient (VDD) diet (25 IU/kg vitamin D3; Xietong, Jiangsu, China) for 9 weeks. Control mice were fed a vitamin D sufficient (VDS) diet (1000 IU/kg vitamin D3; Xietong, Jiangsu, China) that has the same compositions except for vitamin D3 content. To avoid direct ultraviolet light exposure, the mice fed the VDD diet were housed in a dark room. The control VDS mice were kept in a 12-h light/dark cycle. After 9 weeks, both VDS and VDD mice were administered intratracheally with one dose of BLM (1.5 Units/kg) or vehicle. The mice were sacrificed on day 14 or 28 following BLM administration. In some experiments, the mice were treated with losartan at a dose of 1.5 mg/kg/day one day after BLM administration for 28 days. Losartan was dissolved in drinking water. Body weight was monitored daily.

### Lung weight coefficient and wet/dry weight ratio

Mouse lungs were removed immediately after euthanasia, and the wet weight was recorded after the surface blood was aspirated. The lungs were then placed in an incubator at 60 °C for 72 h, and the dry weight was measured. Lung weight coefficient was calculated by dividing the wet lung weight by the mouse body weight. The lung wet/dry weight ratio was calculated by dividing the wet weight by the dry weight of the same lung.

### Histology

Freshly harvested lungs were fixed in 4% formalin made in phosphate-buffered saline for 24 h and embedded in paraffin wax. The tissue blocks were sectioned at 5 μm using a Leica Microtome. Slides were stained with H&E (Beyotime Institute of Biotechnology, Shanghai, China) or Masson’s trichrome (Senbeijia, Nanjing, China) following routine procedures. Microscopic lung fibrosis was scored using the Ashcroft scale^57^, and ECM deposition was quantified with ImageJ (NIH) based on Masson’s trichrome-stained slides. Alveolitis scores were obtained according to a previous study^58^.

### Cell culture and treatment

MLg2908 murine lung fibroblasts were cultured in DMEM supplemented with 10% fetal bovine serum, 100 U/ml penicillin and 100 mg/ml streptomycin at 37 °C and 5% CO_2_. To study dose responses, the cells were treated with different concentrations of TGF-β1 (0, 1, 5, 10 ng/ml) (Sigma-Aldrich, St. Louis, MO) or Ang II (0, 10, 100, 1000 nM) (Sigma-Aldrich, St. Louis, MO) for 24 h. Then cells were treated with 5 ng/ml TGF-β1, 100 nM Ang II or both for 24 h. In some experiments, the cells were pretreated with 1,25(OH)_2_D_3_ (20 nM) or ethanol vehicle for 24 h before TGF-β1 and Ang II co-treatment.

### Western blot

Total protein lysates were extracted from lung tissues or MLg2908 cells by homogenization in the Laemmli sample buffer. An equal amount of proteins (30 μg per lane) were separated by electrophoresis on 8% polyacrylamide gels, and the proteins were electro-transferred onto polyvinylidene difluoride membranes (EMD Millipore, Billerica, MA, USA) overnight. The membranes were then incubated with primary antibodies as follows: anti-β-actin (1:1000, Santa Cruz), anti-α-smooth muscle actin (SMA) (1:1000, CBL171, Millipore), anti-transforming growth factor (TGF)-β1 (1:1000, ab92486, Abcam), anti-collagen type 1 (Col I) (1:1000, ab21286, Abcam), anti-fibronectin (FN) (1:1000, F7387, Sigma-Aldrich), anti-vitamin D receptor (VDR) (1:1000, sc-13133, Santa Cruz), anti-renin (1:1000, sc-133145, Santa Cruz), anti-AT1R (1:1000, ab124734, Abcam) and anti-AGT (1:1000, sc-374511, Santa Cruz). Secondary antibody was horseradish peroxidase-conjugated anti-IgG (ZB-2301, ZB-2305, ZSGB-BIO). The relative amount of proteins in each band was quantified using ImageJ (NIH), and normalized to β-actin (TA-09, ZSGB-BIO) as an internal loading control.

### RT-PCR

Total RNAs were extracted from lung tissues or MLg2908 cells using TRIzol reagent (Invitrogen, Camarillo, CA). First-strand cDNAs were synthesized using PrimeScript RT reagent kit (TaKaRa, Mountain View, CA). Real-time PCR was performed with SYBR Premix Ex kit (TaKaRa, Mountain View, CA) in an ABI 7500 real-time PCR system. The relative amounts of transcripts were calculated using the 2^−ΔΔCt^ formula^59^, and normalized to GAPDH as an internal control. PCR primers used in this study are provided in Table 1.

**Table 1:** Primer sequences used for RT-PCR.

Primer name	Forward (5′–3′)	Reverse (3′–5′)
ma-SMA	GAG GCA CCA CTG AAC CCT AA	CAT CTC CAG AGT CCA GCA CA
mTGF-b1	TGG AGC AAC ATG TGG AAC TCT	CCT GTA TTC CGT CTC CTT GGT
mCol-Ia	GCA GGT TCA CCT ACT CTG TCC T	CTT GCC CCA TTC ATT TGT CT
mCol-IIIa	TCC CCT GGA ATC TGT GAA TC	TGA GTC GAA TTG GGG AGA AT
mCol-IVa	AGG GTT ACC TGG AGA AAA AGG G	TGG TCT CCT TTC TGT CCC TTC
mFN	CGA GGT GAC AGA GAC CAC AA	CTG GAG TCA AGC CAG ACA CA
mRenin	TTT ATC CAC TGA CCC AGT TC	CTG AGA GAA ACC TCT CAT CG
mAT1R	CTG CTC TCC CGG ACT TAA CA	CTG GGT TGA GTT GGT CTC AGA
mAGT	TCT TTG GCA CCC TGG TCT CTT TCT	TTC TCA GTG GCA AGA ACT GGG TCA
mACE1	CCC ATC TGC TAG GGA ACA TGT	GGT GTC CAT CCC TGC TTT ATC A

α-SMA: α-smooth muscle actin, TGF-b1: transforming growth factor-β1, Col-Ia: Collagen I, Col-IIIa: Collagen III, Col-IVa, Collagen IV, FN: fibronectin, AT1R: angiotensin type 1 receptor, AGT: angiotensinogen, ACE1: angiotensin converting enzyme 1.

### Lung lysate assays

Lung lysates were extracted using phosphate-buffered saline (pH 7.0). Concentrations of Ang II, TGF-β1 and tumor necrosis factor (TNF)-α in the lung lysates were determined using Ang II ELISA kit (Nanjing Jiancheng Bioengineering Institute, China), TGF-β1 ELISA kit (ExCell Bio, Taicang, China) and TNF-α ELISA kit (ExCell Bio, Taicang, China) according to the manufacturer’s instructions, respectively. Myeloperoxidase (MPO) activity was assessed using an MPO assay kit (Nanjing Jiancheng Bioengineering Institute, China) according to the manufacturer’s instruction.

### Statistical analysis

Data values were presented as means ± SEM. Normality and homoscedasticity were assessed by Shapiro–Wilk and Square Levene test before applying parametric tests. Two-tailed Student’s *t* test was used for comparing two groups with parametric data; for comparison of multiple groups with parametric data, we performed one-way analysis of variance (ANOVA), two-way ANOVA (for two variable analysis) or two-way repeated measures ANOVA (for weight change analysis). When the data did not pass the normality or homoscedasticity test, we used Kruskal–Wallis one-way ANOVA for the non-parametric test. *P* values < 0.05 were considered statistically significant. Statistical analysis and graphing were carried out using Origin Pro 8.0 (OriginLab Corp, MA).

## Supplementary Information

Supplementary Figures.

## Data Availability

All data generated or analyzed during this study are included in this published article. Data sharing not applicable to this article as no datasets were generated or analyzed during the current study.

## References

[1] Martinez FJ (2017). Idiopathic pulmonary fibrosis. Nat. Rev. Dis. Primers.

[2] Richeldi L, Collard HR, Jones MG (2017). Idiopathic pulmonary fibrosis. Lancet.

[3] Mora AL, Rojas M, Pardo A, Selman M (2017). Emerging therapies for idiopathic pulmonary fibrosis, a progressive age-related disease. Nat. Rev. Drug Discov..

[4] Chanda D (2019). Developmental pathways in the pathogenesis of lung fibrosis. Mol. Aspects Med..

[5] Hutchinson J, Fogarty A, Hubbard R, McKeever T (2015). Global incidence and mortality of idiopathic pulmonary fibrosis: A systematic review. Eur. Respir. J..

[6] Hutchinson JP, McKeever TM, Fogarty AW, Navaratnam V, Hubbard RB (2014). Increasing global mortality from idiopathic pulmonary fibrosis in the twenty-first century. Ann. Am. Thorac. Soc..

[7] Moeller A, Ask K, Warburton D, Gauldie J, Kolb M (2008). The bleomycin animal model: A useful tool to investigate treatment options for idiopathic pulmonary fibrosis?. Int. J. Biochem. Cell Biol..

[8] Bouillon R (2008). Vitamin D and human health: Lessons from vitamin D receptor null mice. Endocr. Rev..

[9] Latimer CS (2014). Vitamin D prevents cognitive decline and enhances hippocampal synaptic function in aging rats. Proc. Natl. Acad. Sci. USA.

[10] Xu S (2015). Vitamin D3 pretreatment regulates renal inflammatory responses during lipopolysaccharide-induced acute kidney injury. Sci. Rep..

[11] Chen L (2019). 1,25-Dihydroxyvitamin D exerts an antiaging role by activation of Nrf2-antioxidant signaling and inactivation of p16/p53-senescence signaling. Aging Cell.

[12] Chaiprasongsuk A (2019). Protective effects of novel derivatives of vitamin D(3) and lumisterol against UVB-induced damage in human keratinocytes involve activation of Nrf2 and p53 defense mechanisms. Redox Biol..

[13] Li YC (2002). 1,25-Dihydroxyvitamin D(3) is a negative endocrine regulator of the renin–angiotensin system. J. Clin. Invest..

[14] Paul M, Poyan Mehr A, Kreutz R (2006). Physiology of local renin–angiotensin systems. Physiol. Rev..

[15] Phillips MI, Kagiyama S (2002). Angiotensin II as a pro-inflammatory mediator. Curr. Opin. Investig. Drugs.

[16] Uhal BD, Li X, Piasecki CC, Molina-Molina M (2012). Angiotensin signalling in pulmonary fibrosis. Int. J. Biochem. Cell Biol..

[17] Specks U, Martin WJ, Rohrbach MS (1990). Bronchoalveolar lavage fluid angiotensin-converting enzyme in interstitial lung diseases. Am. Rev. Respir. Dis..

[18] Selman M (2006). Gene expression profiles distinguish idiopathic pulmonary fibrosis from hypersensitivity pneumonitis. Am. J. Respir. Crit. Care Med..

[19] Wang J (2015). Chronic activation of the renin–angiotensin system induces lung fibrosis. Sci. Rep..

[20] Wang Y, Zhu J, DeLuca HF (2012). Where is the vitamin D receptor?. Arch. Biochem. Biophys..

[21] Belderbos ME (2011). Cord blood vitamin D deficiency is associated with respiratory syncytial virus bronchiolitis. Pediatrics.

[22] Łuczyńska A (2014). Cord blood 25(OH)D levels and the subsequent risk of lower respiratory tract infections in early childhood: The Ulm birth cohort. Eur. J. Epidemiol..

[23] Dancer RC (2015). Vitamin D deficiency contributes directly to the acute respiratory distress syndrome (ARDS). Thorax.

[24] Vanstone MB, Egan ME, Zhang JH, Carpenter TO (2015). Association between serum 25-hydroxyvitamin D level and pulmonary exacerbations in cystic fibrosis. Pediatr. Pulmonol..

[25] Tzilas V (2019). Vitamin D prevents experimental lung fibrosis and predicts survival in patients with idiopathic pulmonary fibrosis. Pulm. Pharmacol. Ther..

[26] Shi Y (2017). Chronic vitamin D deficiency induces lung fibrosis through activation of the renin–angiotensin system. Sci. Rep..

[27] Kong J (2013). VDR attenuates acute lung injury by blocking Ang-2-Tie-2 pathway and renin–angiotensin system. Mol. Endocrinol..

[28] Zhang Z (2015). Preventive effects of vitamin D treatment on bleomycin-induced pulmonary fibrosis. Sci. Rep..

[29] Wynn TA (2011). Integrating mechanisms of pulmonary fibrosis. J. Exp. Med..

[30] Gross TJ, Hunninghake GW (2001). Idiopathic pulmonary fibrosis. N. Engl. J. Med..

[31] Liu YM, Nepali K, Liou JP (2017). Idiopathic pulmonary fibrosis: Current status, recent progress, and emerging targets. J. Med. Chem..

[32] Noble PW (2003). Idiopathic pulmonary fibrosis. New insights into classification and pathogenesis usher in a new era therapeutic approaches. Am. J. Respir. Cell Mol. Biol..

[33] Selman M, Pardo A (2003). The epithelial/fibroblastic pathway in the pathogenesis of idiopathic pulmonary fibrosis. Am. J. Respir. Cell Mol. Biol..

[34] Plataki M (2005). Expression of apoptotic and antiapoptotic markers in epithelial cells in idiopathic pulmonary fibrosis. Chest.

[35] Tashiro J (2017). Exploring animal models that resemble idiopathic pulmonary fibrosis. Front. Med. (Lausanne).

[36] Segel MJ (2005). Effect of IL-2-Bax, a novel interleukin-2-receptor-targeted chimeric protein, on bleomycin lung injury. Int. J. Exp. Pathol..

[37] Berkman N (2001). Human recombinant interferon-alpha2a and interferon-alphaA/D have different effects on bleomycin-induced lung injury. Respiration.

[38] Silver RB (2004). Mast cells: a unique source of renin. Proc. Natl. Acad. Sci. USA.

[39] Szeto FL (2012). Vitamin D receptor signaling inhibits atherosclerosis in mice. Mol. Endocrinol..

[40] Veerappan A (2008). Mast cell renin and a local renin–angiotensin system in the airway: Role in bronchoconstriction. Proc. Natl. Acad. Sci. USA.

[41] Marshall RP (2004). Angiotensin II and the fibroproliferative response to acute lung injury. Am. J. Physiol. Lung Cell. Mol. Physiol..

[42] Murphy AM, Wong AL, Bezuhly M (2015). Modulation of angiotensin II signaling in the prevention of fibrosis. Fibrogenesis Tissue Repair..

[43] Jiang JS (2012). Activation of the renin–angiotensin system in hyperoxia-induced lung fibrosis in neonatal rats. Neonatology.

[44] Bernstein CN, Bector S, Leslie WD (2003). Lack of relationship of calcium and vitamin D intake to bone mineral density in premenopausal women with inflammatory bowel disease. Am. J. Gastroenterol..

[45] Holick MF (2006). Resurrection of vitamin D deficiency and rickets. J. Clin. Invest..

[46] Qiao G, Kong J, Uskokovic M, Li YC (2005). Analogs of 1alpha,25-dihydroxyvitamin D(3) as novel inhibitors of renin biosynthesis. J. Steroid Biochem. Mol. Biol..

[47] Kong J, Qiao G, Zhang Z, Liu SQ, Li YC (2008). Targeted vitamin D receptor expression in juxtaglomerular cells suppresses renin expression independent of parathyroid hormone and calcium. Kidney Int..

[48] Yuan W (2007). 1,25-Dihydroxyvitamin D3 suppresses renin gene transcription by blocking the activity of the cyclic AMP response element in the renin gene promoter. J. Biol. Chem..

[49] Hagaman JT (2011). Vitamin D deficiency and reduced lung function in connective tissue-associated interstitial lung diseases. Chest.

[50] Finklea JD, Grossmann RE, Tangpricha V (2011). Vitamin D and chronic lung disease: A review of molecular mechanisms and clinical studies. Adv. Nutr..

[51] Førli L (2004). Vitamin D deficiency, bone mineral density and weight in patients with advanced pulmonary disease. J. Intern. Med..

[52] Udomsinprasert W, Jittikoon J (2019). Vitamin D and liver fibrosis: Molecular mechanisms and clinical studies. Biomed. Pharmacother..

[53] de Bragança AC (2018). Vitamin D deficiency aggravates the renal features of moderate chronic kidney disease in 5/6 nephrectomized rats. Front. Med. (Lausanne).

[54] Tao Q (2015). Vitamin D prevents the intestinal fibrosis via induction of vitamin D receptor and inhibition of transforming growth factor-beta1/Smad3 pathway. Dig. Dis. Sci..

[55] Zhang Y, Kong J, Deb DK, Chang A, Li YC (2010). Vitamin D receptor attenuates renal fibrosis by suppressing the renin–angiotensin system. J. Am. Soc. Nephrol..

[56] U.S. Cancer Statistics Working Group. United States Cancer Statistics: 1999–2013 Incidence and Mortality Web-based Report. Atlanta, GA: U.S. Department of Health and Human Services, Centers for Disease Control and Prevention and National Cancer Institute (2016). www.cdc.gov/uscs.

[57] Hübner, R. H. *et al.* Standardized quantification of pulmonary fibrosis in histological samples. *Biotechniques* **44**, 507–511, 514–507. 10.2144/000112729 (2008) 10.2144/000112729 18476815

[58] Szapiel SV, Elson NA, Fulmer JD, Hunninghake GW, Crystal RG (1979). Bleomycin-induced interstitial pulmonary disease in the nude, athymic mouse. Am. Rev. Respir. Dis..

[59] Schmittgen TD, Livak KJ (2008). Analyzing real-time PCR data by the comparative C(T) method. Nat. Protoc..

