# Supplementary material for: Vitamin D suppresses bleomycin-induced pulmonary fibrosis by targeting the local renin–angiotensin system in the lung
Source: Sci Rep. 2021 Aug 16;11:16525. doi: 10.1038/s41598-021-96152-7 (PMC8367953; doi:10.1038/s41598-021-96152-7)

Figure 2B

α-SMA

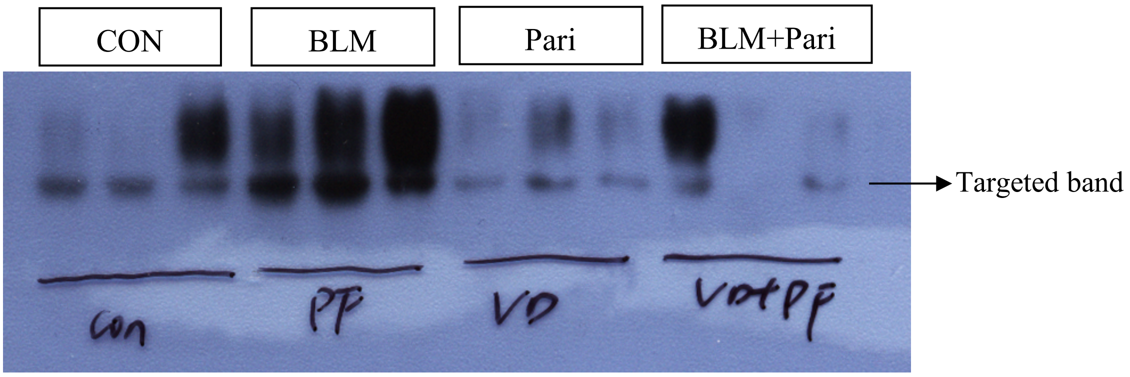

TGF-β1

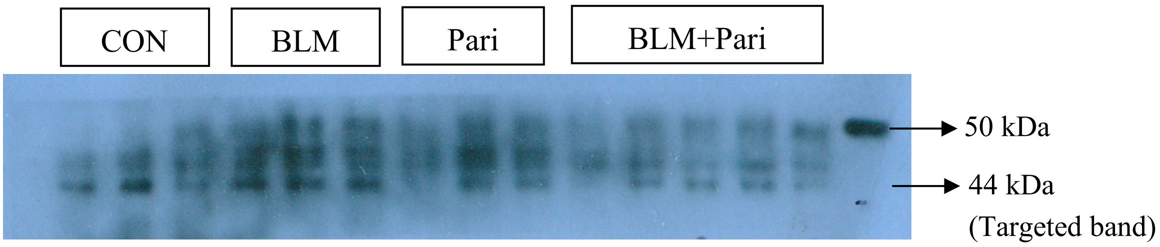

Col I

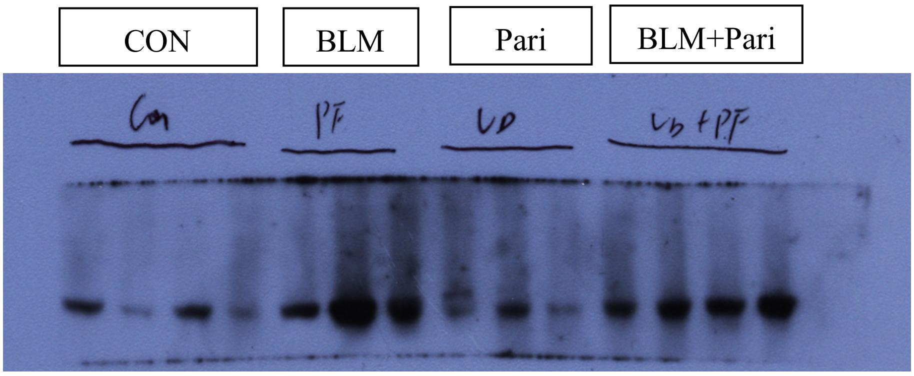

FN

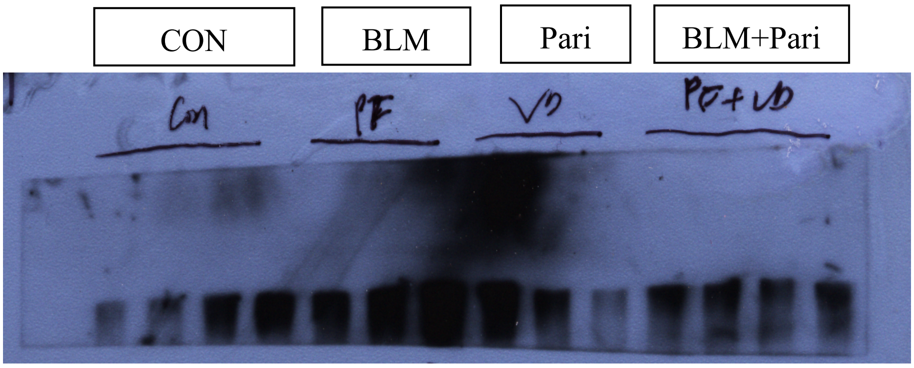

β-actin

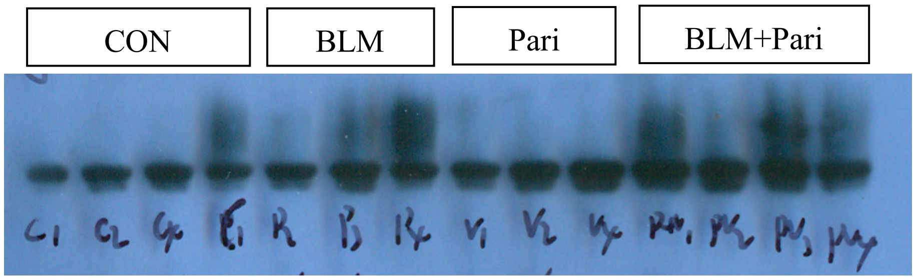

Figure 3B

VDR

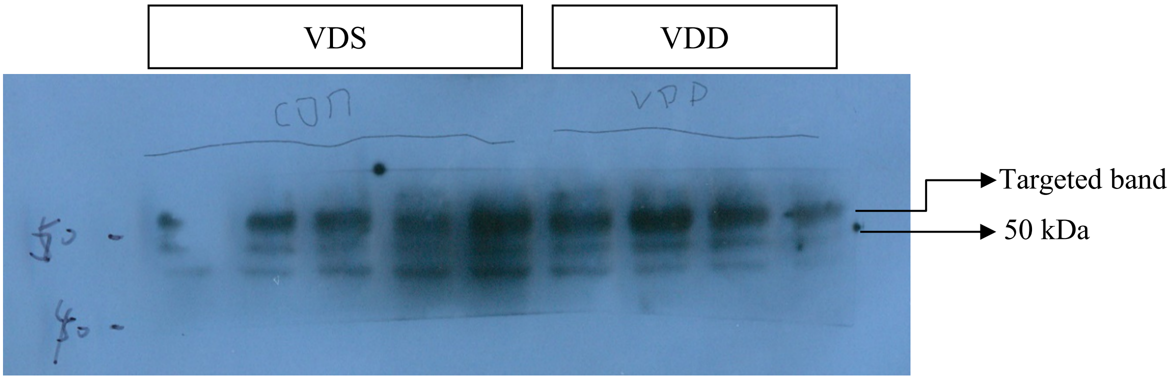

β-actin

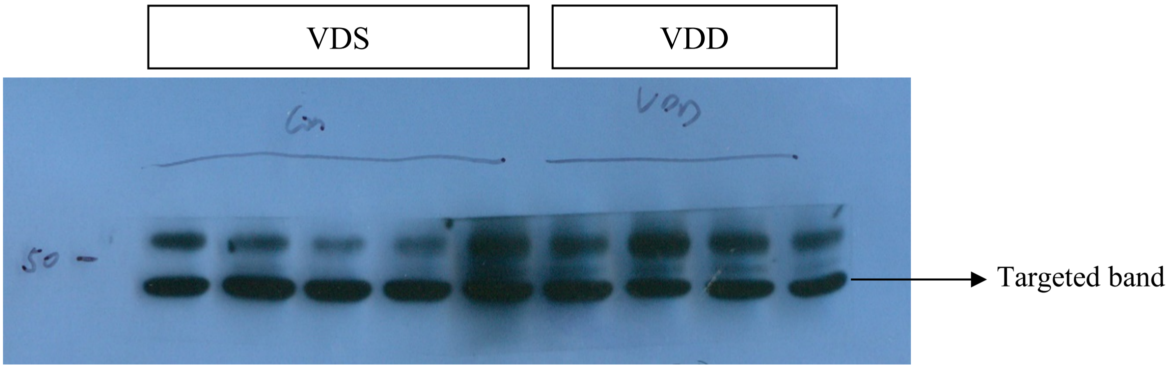

Figure 4B

α-SMA

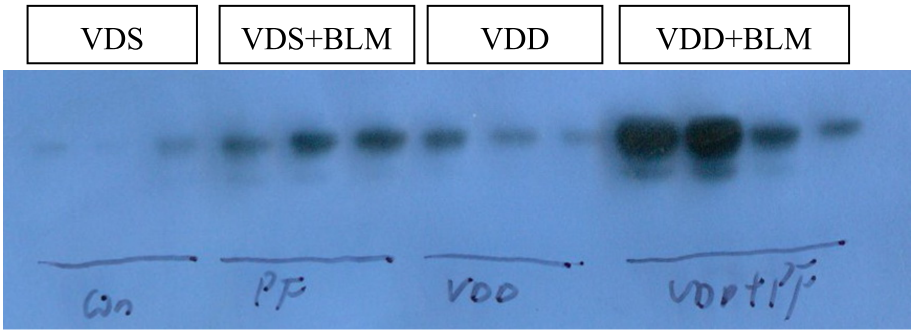

TGF-β1

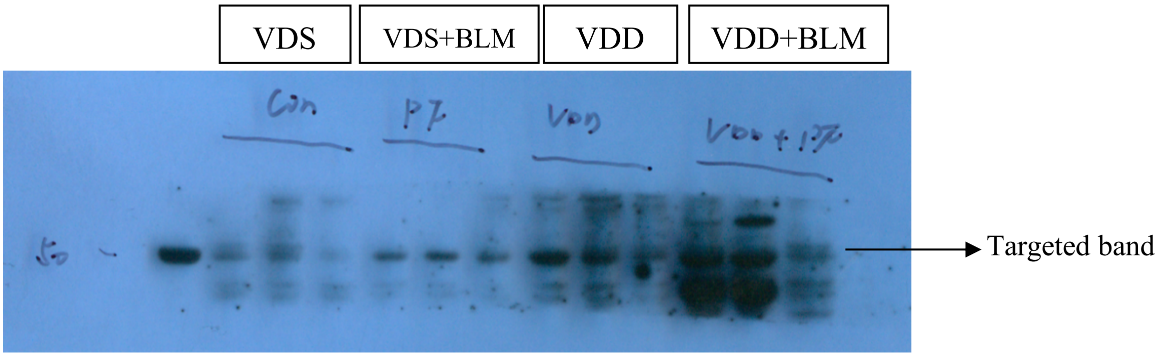

Col I

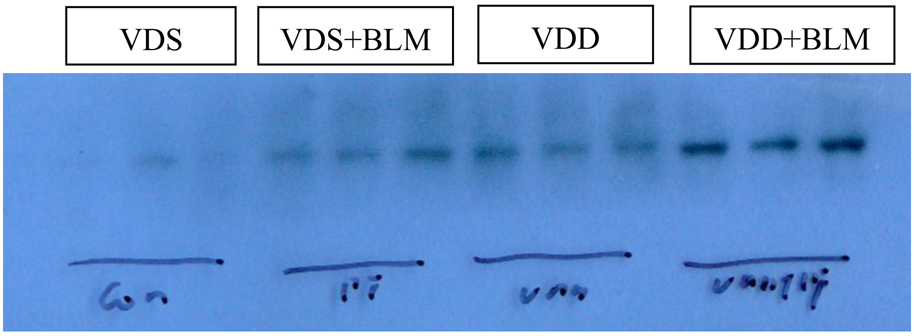

FN

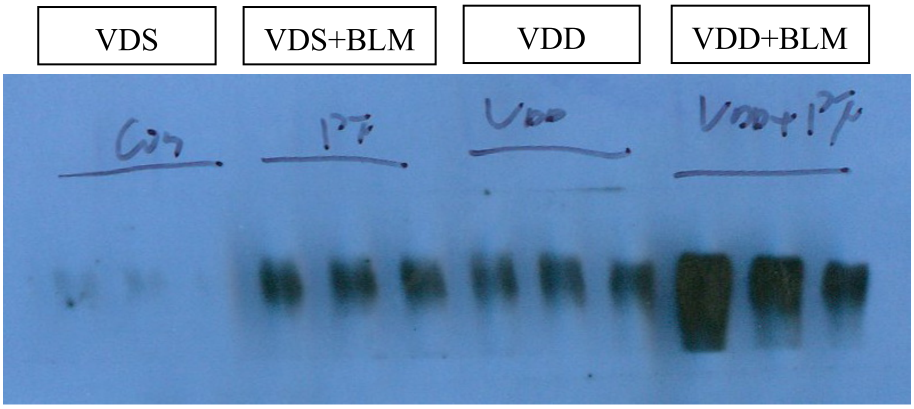

β-actin

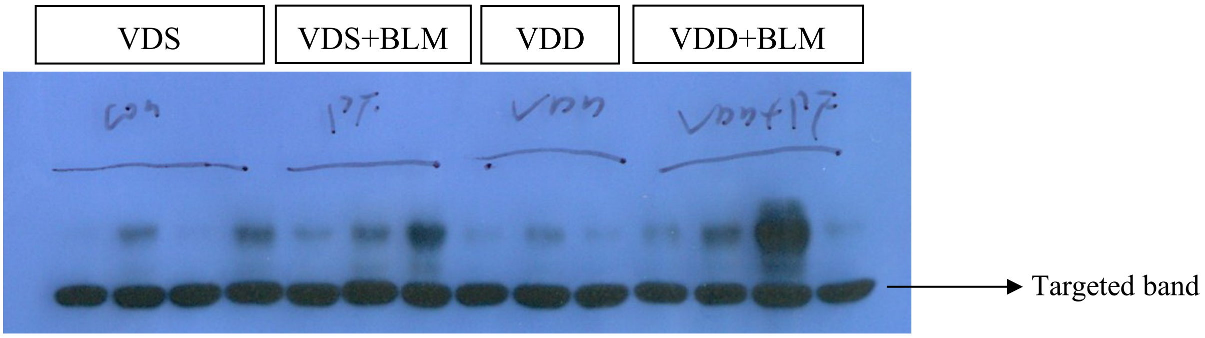

Figure 5B

Renin

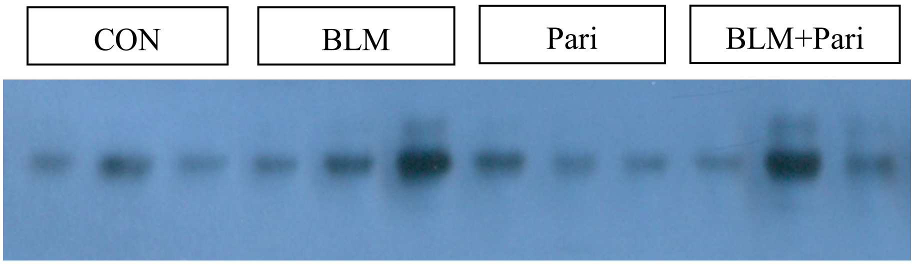

AT1R

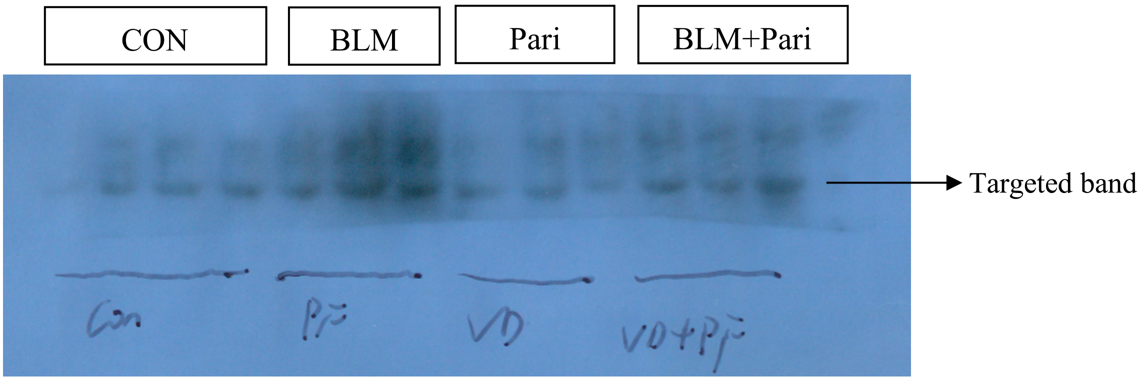

AGT

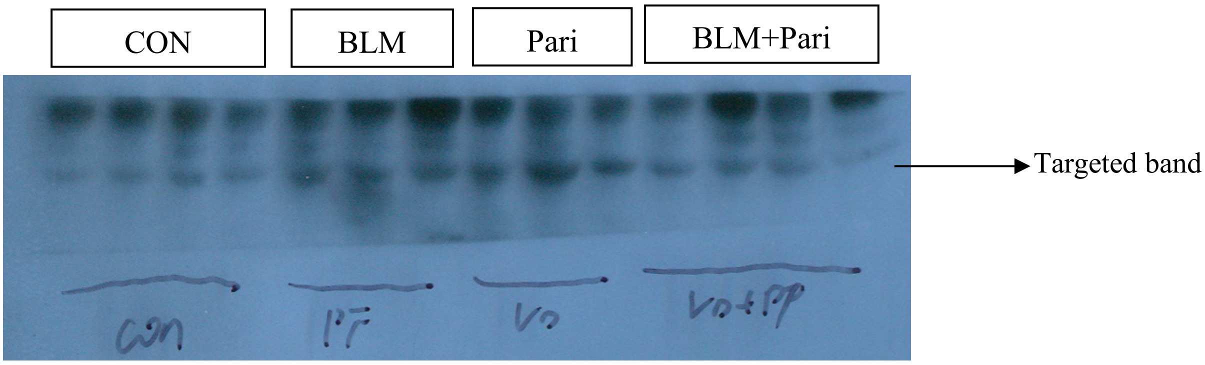

β-actin

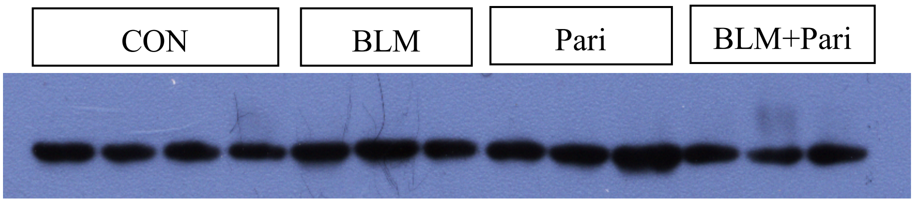

Figure 5E

Renin

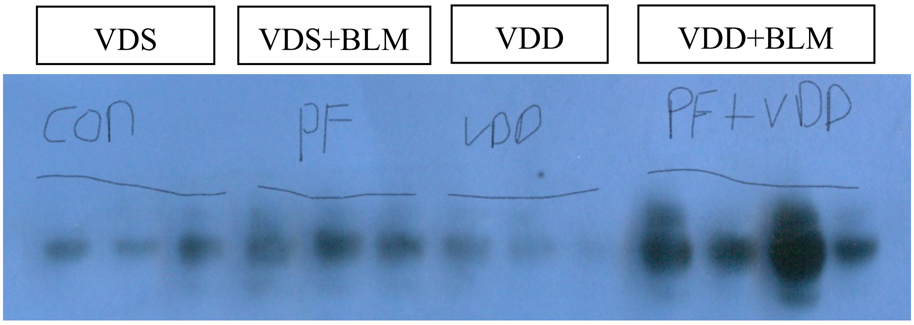

AT1R

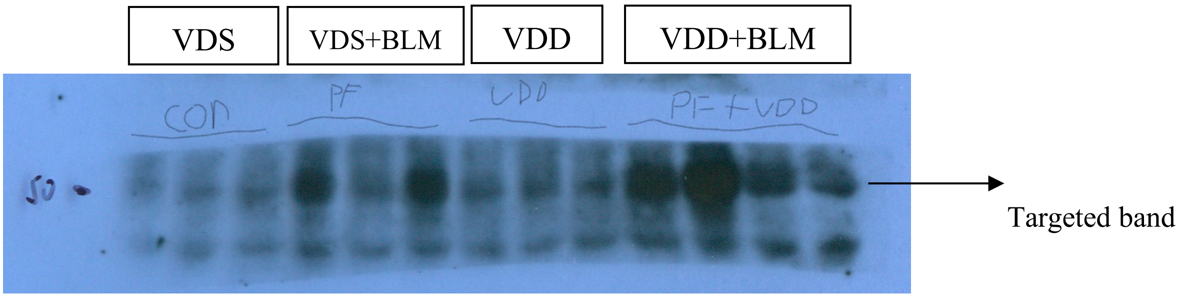

AGT

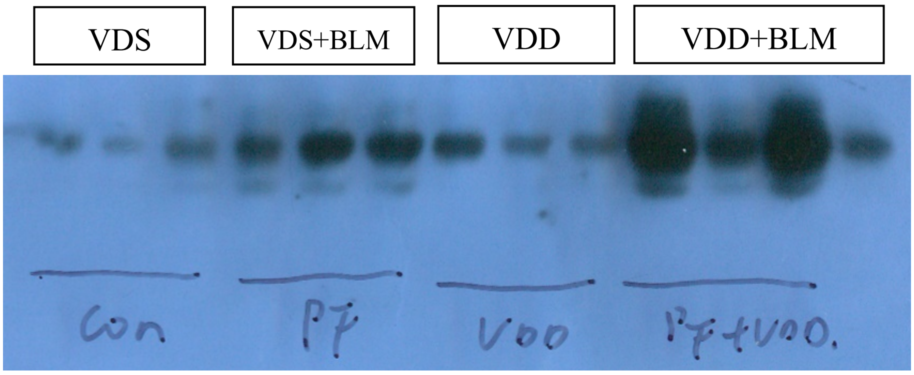

β-actin

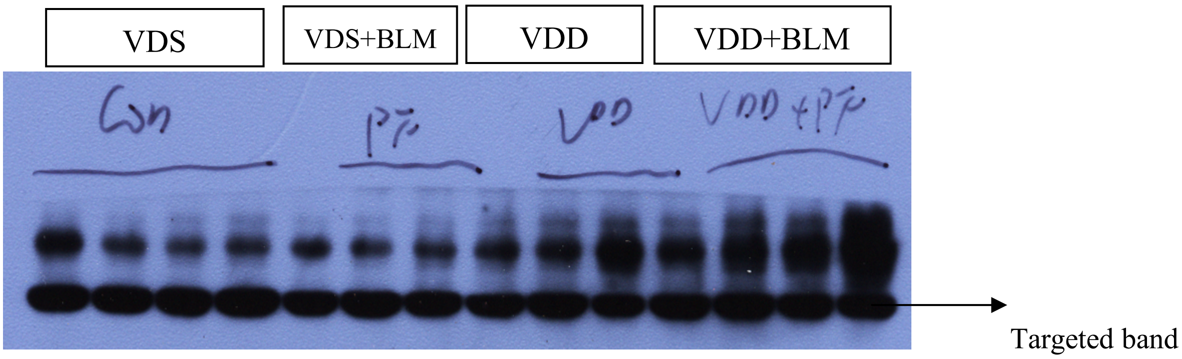

Figure 7A

α-SMA

TGF-β1 (ng/ml)

0 1 5 10

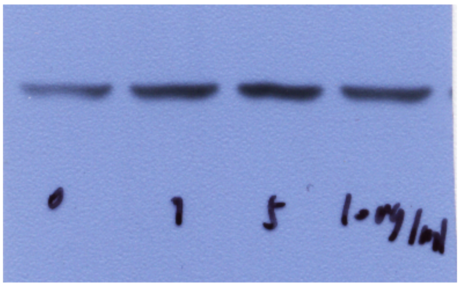

β-actin

TGF-β1 (ng/ml)

0 1 5 10

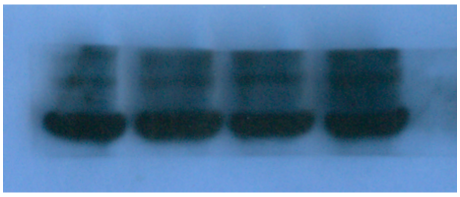

Figure 7C

α-SMA

Ang Ⅱ (nM)

0 10 100 1000

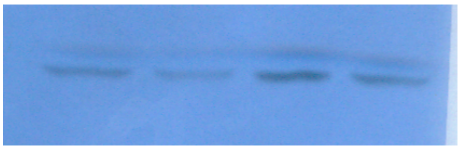

β-actin

Ang Ⅱ (nM)

0 10 100 1000

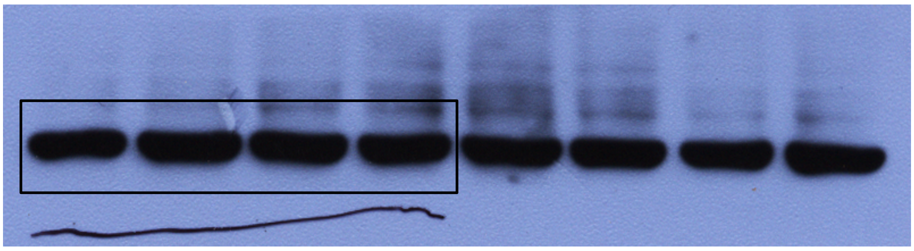

Figure 7E

AT1R

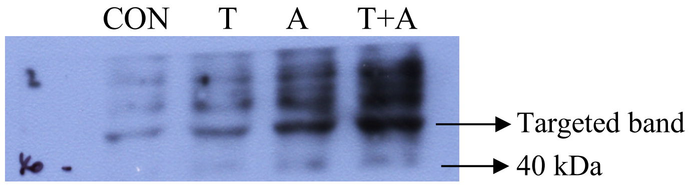

α-SMA

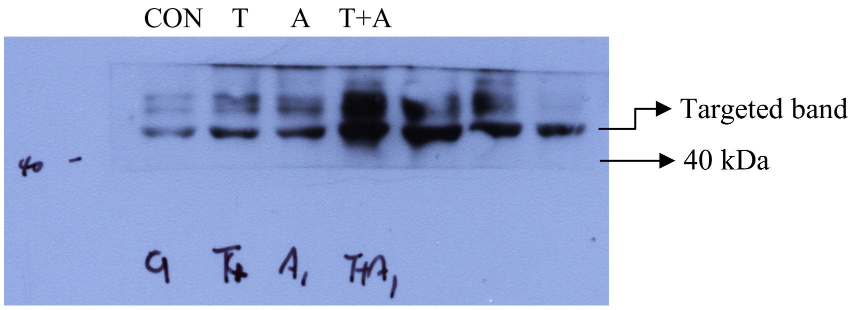

TGF-β1

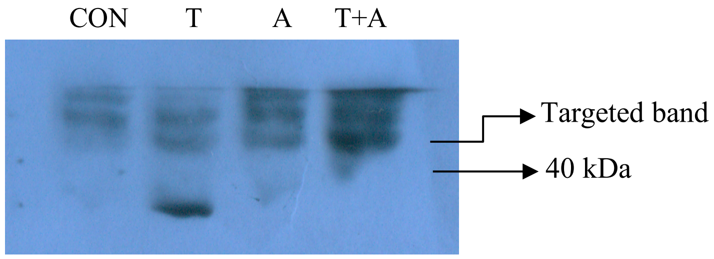

Col I

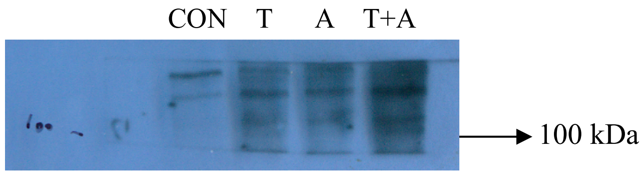

FN

CON T A T+A

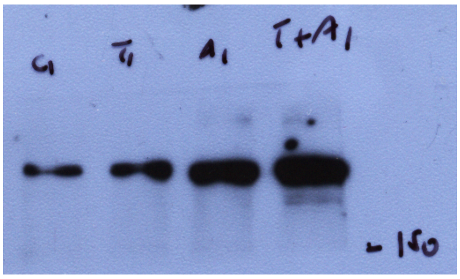

β-actin

CON T A T+A

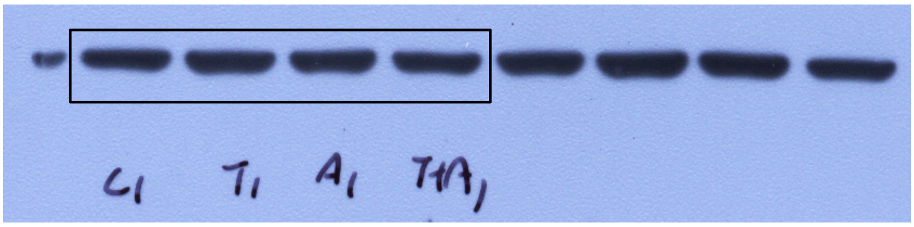

Figure 7G

α-SMA

CON VD T+A T+A+VD

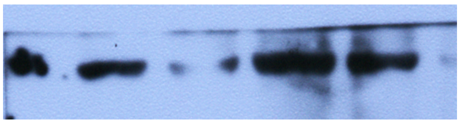

TGF-β1

CON VD T+A T+A+VD

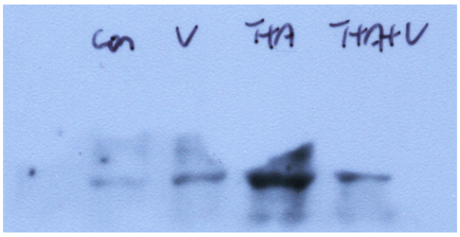

Col I

CON VD T+A T+A+VD

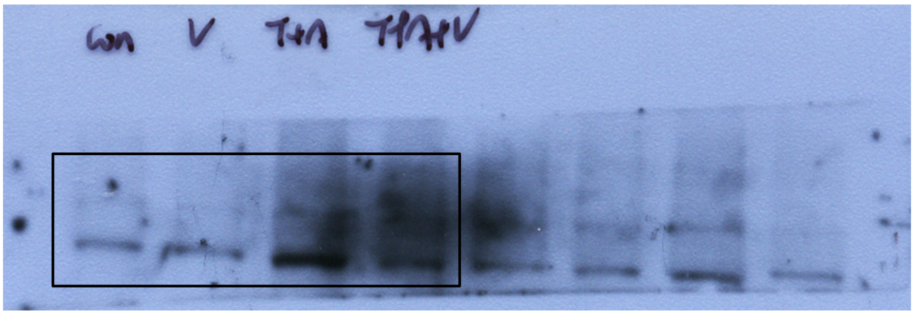

FN

CON VD T+A T+A+VD

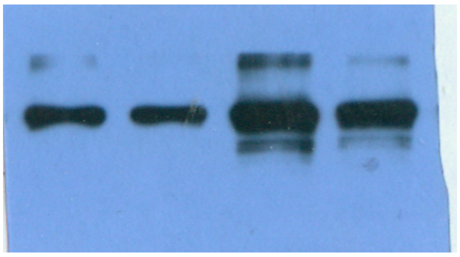

β-actin

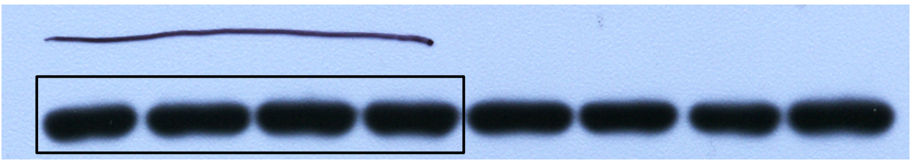

Supplement: Supplementary file 1 — Supplementary Figures. [file 41598_2021_96152_MOESM1_ESM.pdf]
